# The development of a serious game for laser applications in dentistry and the evaluation of dental students' satisfaction

**DOI:** 10.1186/s12909-024-05563-3

**Published:** 2024-05-28

**Authors:** Maryam Khorasanchi, Melika Hoseinzadeh, Majid Khadem Rezaeian, Ali Kazemian, Ali Moradi, Javad Sarabadani

**Affiliations:** 1 Department of Endodontics, School of Dentistry, Mashhad University of Medical Sciences, Mashhad, Iran; 2 Dentist, Research Assistant, Dental Research Center, Mashhad Dental School, Mashhad University of Medical Sciences, Mashhad, Iran; 3 Department of Community Oral Health, Faculty of Dentistry, Mashhad University of Medical Sciences, Mashhad, Iran; 4 Department of Community Medicine and Public Health, Faculty of Medicine, Mashhad University of Medical Sciences, Mashhad, Iran; 5 Orthopedic Research Center, Mashhad University of Medical Science, Mashhad, Iran; 6 Bone and Joint Research Laboratory, Ghaem Hospital, Mashhad University of Medical Science, Mashhad, Iran; 7 Oral and Maxillofacial Disease Research Center, School of Dentistry, Mashhad University of Medical Sciences, Mashhad, Iran

**Keywords:** Educational technology, Gamification, e-learning, Dental education, Laser

## Abstract

**Background:**

This study aimed to design and implement a gamified application about the theoretical aspects of laser applications in dentistry and investigate students' satisfaction with their learning experience.

**Methods:**

An engaging educational program named Essential Skills and Knowledge in Learning Laser (ESKILLD) was developed to teach laser applications in dentistry. The program comprises two primary components: a “Tutorial and Quiz” section and a “Games” section. Final-year dental students were tasked with installing and using this application. A 29-item validated questionnaire (Cronbach's alpha = 0.97, ICC = 0.94) was used to evaluate students' perceptions of the applications' design and functionality. The influence of participants' gender and Grade Point Average (GPA) on their satisfaction levels was examined via the student t-test and Pearson's correlation, with a significance level of 0.05.

**Results:**

The study had 56 participants, of which 37 were female (66.07%), and 19 were male (33.92%). The students' average GPA was 15.16 out of 20. The mean rating for ESKILLD's design and functionality was 1.39 ± 0.47 and 1.37 ± 0.46, respectively, signifying a high satisfaction level. Female students rated the application's coherence and learning perception significantly higher than their male counterparts (*p* < 0.05). However, gender did not significantly influence scores on other perception aspects or overall scores. Students' GPAs and their perception scores did not have a significant correlation.

**Conclusions:**

The results indicate that the participants were generally satisfied with the game's features and attitudes towards it, which underscores the potential effectiveness of gamification in dental courses focused on laser applications.

## Introduction

Laser technology has been introduced into the dental field to shorten treatment times, increase treatment efficiency and effectiveness, and improve patient comfort. It has been demonstrated that lasers can improve wound healing and reduce postoperative complications, blood loss, and trauma at the surgical site [1]. Lasers can be used for incision, ablation, tissue healing, the removal of hyperplastic tissue, and photodynamic therapy [2]. Due to the growing popularity of laser dentistry, it is necessary to educate dental students about this subject properly.

In Iran, traditional dental education includes didactic courses, preclinical lab training, and clinical patient experiences, similar to other countries. Over the last two decades, advances in educational approaches have provided new methods to explore and improve students' performance. Technological advancements and the widespread use of digital devices have allowed the incorporation of E-learning into educational systems [3]. Smartphone-based mobile learning has a comparable, and even slightly superior, efficacy to lecture-based learning [4].

Gamification incorporates game elements into non-game contexts to increase student motivation and engagement [5]. Game-based learning has a pedagogical purpose [6]. In dentistry, the use of online gamification platforms, such as Kahoot!®, has resulted in increased satisfaction among dental students in the teaching of microbiology, immunology, and histology concepts [3, 7]. Game-based learning has been effective for various subjects in dentistry, including oral histology [6], orthodontics [8], learning orofacial spaces [9], oral lesions diagnosis and treatment planning [10], endodontics [11], oral rehabilitation of edentulous patients [12], clinical communication [13], teledentistry training [14], basic life support skills [15], and clinical reasoning skills [16], has shown a positive effect on students' motivation, self-perceived confidence, awareness, self-efficacy in learning, cognitive skills, knowledge. Digital gamification platforms engage students with different learning styles due to their easy accessibility and ability to integrate well with varied teaching methods while significantly increasing student participation and engagement in the classroom compared to traditional lecture-based teaching [17]. Furthermore, gamification can enhance the learning experience and course satisfaction by providing joy and excitement through educational games, self-assessment, and rapid feedback [3].

The satisfaction of students with their learning experience is linked to improved mental and physical health as well as academic success [18]. Due to the benefits of game-based learning, the current study investigated dental students' satisfaction with a newly developed gamified mobile application as an adjunctive method in educating laser applications in dentistry. Additionally, the study examined the relationship between students' satisfaction and their demographic characteristics to determine if the game could be beneficial for students with different genders and educational backgrounds.

## Method and material

### Setting and population

The present cross-sectional study was conducted at the Mashhad University of Medical Sciences Dental School from September 2021 to June 2022. The study was conducted in two phases: designing the game and evaluating students’ satisfaction with the application feathers and their learning experience.

The school curriculum for Laser Applications in Dentistry includes one theoretical and one practical course for 6th-year students, namely Laser in Dentistry and Clinical Applications of Laser in Dentistry, respectively. The students who were taking the courses during the study voluntarily participated in the study through total population sampling.

### Game design

An educational and entertaining application for laser application in dentistry was designed and created for the Android operating system. The application was named ESKILLD, a synonym for **E**ssential **S**kills and **K**nowledge **i**n **L**earning **L**aser. The initial development of the application was based on focus group interviews conducted with a group of three undergraduate students, a post-graduate student (M.K.), a professor of Oral and Maxillofacial Diseases (J.S.), a professor of Community Oral Health (A.K.), a professor of Community Medicine and Public Health (M. KR.), and a software developer and media designer. The topics investigated in the focus group were framework conditions, gamification aspects, digital platforms, and possible applications. The instructor (J.S.) translated the content into English, and certified translators translated it into French and Italian. The final version of the application was developed using Ktor, an open-source framework for the Kotlin programming language, which is available for Android operating systems. The entire development process took approximately three months with an estimated cost of $560.

**Fig. 1:**
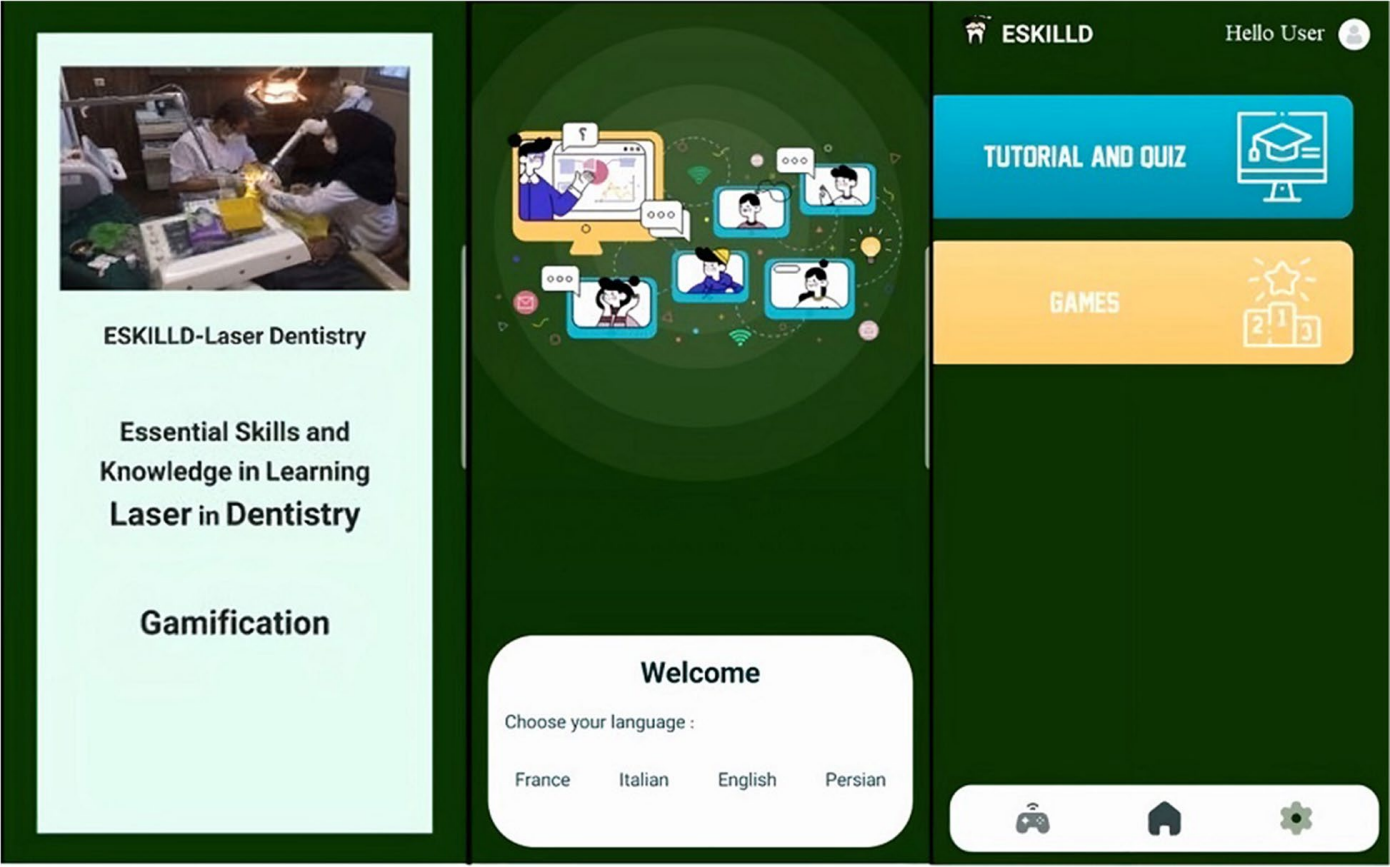
After registration, learners chose their preferred language from Persian, English, French, and Italian. Then, they selected "Tutorial and Quiz" or "Games"

**Fig. 2:**
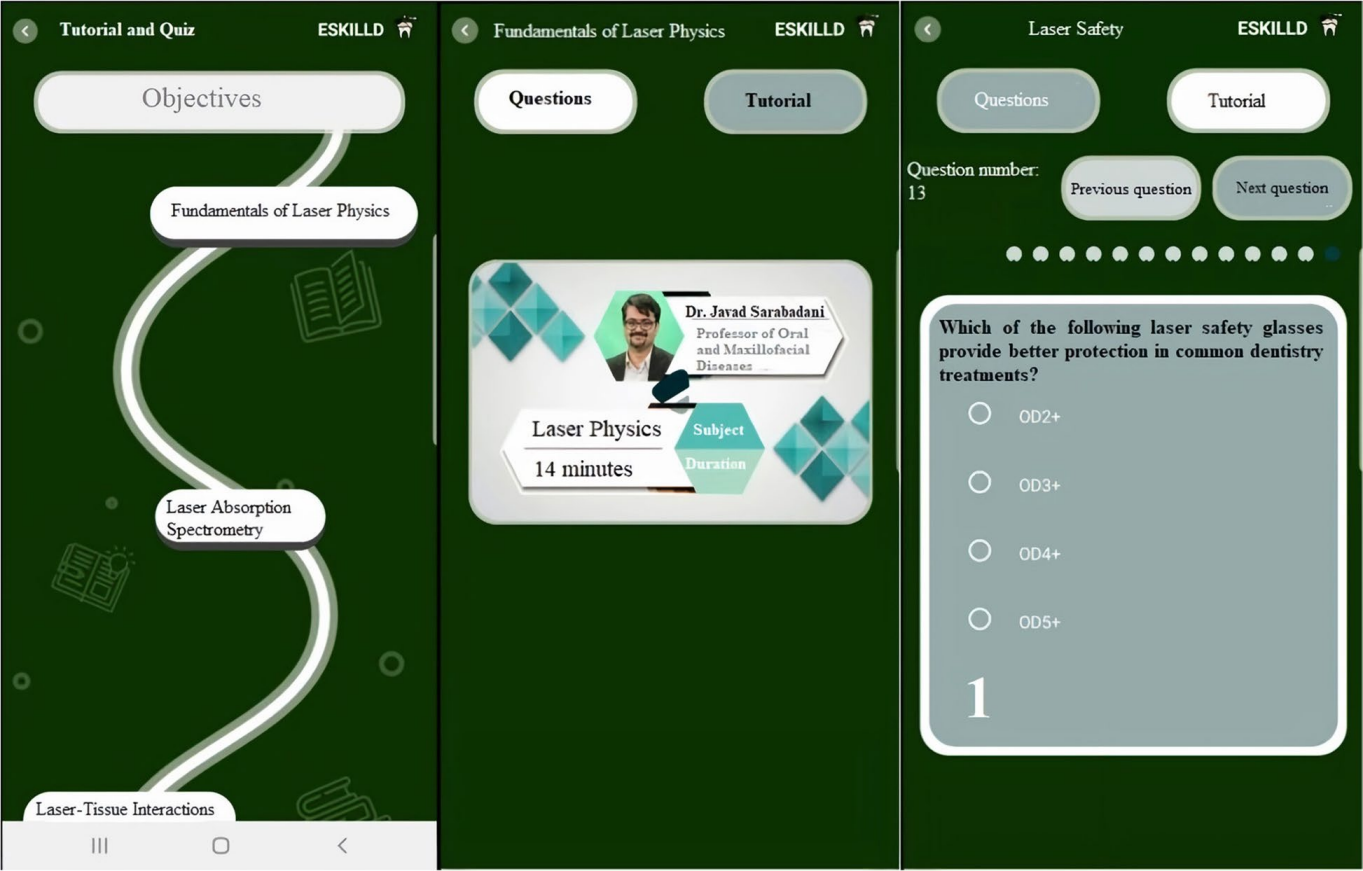
When the students selected "Tutorial and Quiz," they were guided through the objectives on a "road map." Then, they selected the subject, watched the tutorial, and answered the questions about that subject

**Fig. 3:**
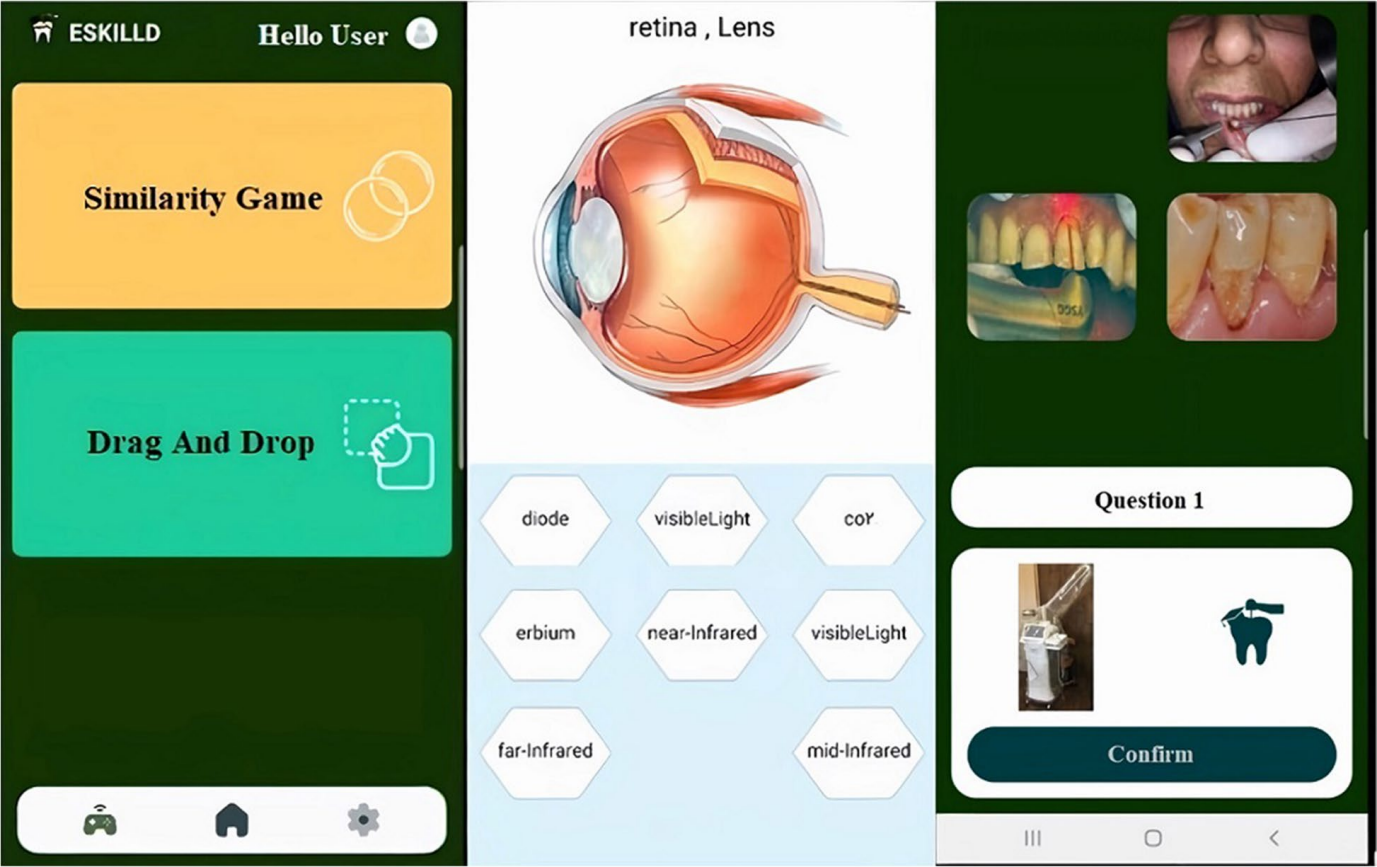
After selecting "Games," the students decided to play a "Drag-and-drop game" for laser safety or a "Similarity game" for selecting clinical applications related to different types of lasers

Learners first needed to registrar in the application and choose their preferred language from Persian, English, French, and Italian (Fig. 1). After selecting the language, students were provided the option to select “Tutorial and Quiz” or “Games” (Fig. 1):

A **Tutorial and Quiz** (Fig. 2): The user is guided through six objectives in this part, following a “road map.” These objectives correspond to the Laser in Dentistry course curriculum and include: 1) Fundamentals of Laser Physics, 2) Laser Absorption Spectrometry, 3) Laser-Tissue Interactions, 4) Laser Safety, 5) General Applications of Lasers in Dentistry, and 6) Unique Applications of Lasers in Dentistry. Each objective contained short educational videos with subtitles recorded by the instructor (J.S.). The video's duration ranged from 6 to 20 min. Several questions from the subjects followed the videos. Several questions from the subjects followed the videos. According to the feedback system within the game, learners could receive immediate responses.
B **Games** (Fig. 3): When participants chose the “Games” section, they could opt for a “Drag-and-drop game” to learn about laser safety or a “Similarity game” to assess students' understanding of different types of lasers and their clinical applications by selecting related pictures.

The application's domain (www.eskilld.ir) had a panel manager. The instructor could register on the panel manager and monitor students' performance regarding time spent on the application and correct and incorrect answers to the questions.

### Students’ satisfaction

The satisfaction of the participants with their learning experience was evaluated using a researcher-made online questionnaire consisting of 29 items adapted from previous literature. The questionnaire was prepared to reflect the games' design and performance with nine perceptions: Presentation, Attraction, Coherency, Esthetic and Consistency, Visual Aspects of Pictures, Text and Content, Technology and Multimedia Support, Educational Aspects of the Content, Attitude, and Learning.

The content validity of the questionnaire was determined using the Content Validity Index (CVI) and Content Validity Ratio (CVR) [19]. A group of 10 experts specializing in Oral and Maxillofacial Diseases individually assessed each questionnaire item for relevance and clarity. Experts rated the relevance of each item on a scale from 1 (not relevant) to 4 (highly relevant) and the essentiality of each item on a scale from 1 (essential) to 3 (not essential). The CVR was calculated using the formula:

$$CVR=\frac{(Ne-N/2)}{N/2}$$

Ne represents the number of experts rated the item as “Essential,” and N is the total number of experts.

The CVI was calculated by dividing the number of experts who rated each item as either 3 or 4 in terms of relevance by the total number of experts. In this study, the CVR and CVI values were 0.64 and 0.80, respectively, indicating satisfactory content validity.

The questionnaire's internal consistency was assessed using Cronbach's alpha coefficient. Data from 20 dental students resulted in a Cronbach's alpha value of 0.97, indicating excellent internal consistency among the questionnaire items.

Test–retest reliability was evaluated by administering the questionnaire to a subgroup of 20 dental students on two occasions, two weeks apart. Analysis using the Intraclass Correlation Coefficient (ICC) showed strong agreement between the two administrations, with an ICC value of 0.94, demonstrating the high stability of the questionnaire over time.

The instructor, J.S., conducted a 30-min in-person demonstration of the application in the Laser department. Students were asked to install the application on their cell phones and use it along with the course and traditional lectures. Six students did not have access to an Android operating system. Following the in-person demonstration, they were given a cell phone with an Android operating system to try out the application in that session, and they were allowed to go to the Laser department and use the application whenever they wanted to.

Participants were asked to rank the satisfaction survey questions according to a 5-point Likert-type scale ("Strongly agree = 2," "Agree = 1," "Neither agree nor disagree = 0," "Disagree = -1," "Strongly disagree = -2"). The final scores were interpreted as follows: 1 to 2 for excellent, 0 to 1 for good, 0 to -1 for poor, and -1 to -2 for very poor. Additionally, demographic data including participants' gender and the grade point average (GPA) from their preceding five years of education were recorded.

### Statistical analysis

Data analysis was conducted using SPSS software version 23 (IBM, NY, US). To evaluate the association of participants’ gender with their satisfaction level was evaluated using the student t-test. Participants’ GPA correlation with their satisfaction level were evaluated by Pearson’s correlation (α = 0.05).

### Ethical considerations

The protocol of the present study was approved by the Research and Ethics Committee of Mashhad University of Medical Sciences (IR.MUMS.DENTISTRY.REC.1400.031). Participants had the right to withdraw from the study whenever they desired. Assurance was given that their identity would remain confidential and the study results would be published anonymously.

## Results

Fifty-six students participated in the study, including 37 female (66.07%) and 19 male (33.92%) students. The participants' mean age was 24.96 ± 2.79 years old, and their mean GPA score was 15.16 ± 4.86/20.

Table 1 represents the student's scores in each item of the satisfaction questionnaire. The mean score for Presentation (1.48 ± 0.47), Attraction (1.35 ± 0.48), Coherency (1.39 ± 0.59), Esthetic and Consistency (1.34 ± 0.64), Visual Aspects of Pictures, Text and Content (1.28 ± 0.61), Technology and Multimedia Support (1.49 ± 0.60), Educational Aspects of the Content (1.26 ± 0.58), Attitude (1.40 ± 0.61), Learning (1.38 ± 0.66) and Total Score (1.37 ± 0.46) were between in the 1–2 range, which was interpreted as excellent (Table 1).

**Table 1:** Survey questions and mean score for each question and presentation

Presentation	Questions		Strongly agree	Agree	Neither agree nor disagree	Disagree	Strongly disagree	Mean ± SD	Mean ± SD of perceptions
Presentation	1	I think the language used in the game is straightforward and fluent	36 (64.3)	18 (32.10)	0 (0.00)	2 (3.6)	0 (0.00)	1.57 ± 0.68	1.48 ± 0.47
	2	I think, the questions and environment match the images	29 (51.8)	26 (46.4)	1 (1.8)	0 (0.00)	0 (0.00)	1.50 ± 0.53	
	3	The instructional videos, in my opinion, are appropriate for the course's objectives	35 (62.5)	19 (33.9)	2 (3.6)	0 (0.00)	0 (0.00)	1.59 ± 0.56	
	4	The game's content, in my opinion, is appropriate for teaching how to use laser devices	29 (51.8)	24 (42.9)	2 (3.6)	1 (1.8)	0 (0.00)	1.45 ± 0.65	
	5	In my opinion, the game designer used a variety of media (audio and video) to present the content	26 (46.4)	22 (39.3)	7 (12.5)	1 (1.8)	0 (0.00)	1.30 ± 0.76	
Attraction	6	I think the game's content is interesting	19 (33.9)	29 (51.8)	8 (14.3)	0 (0.00)	0 (0.00)	1.20 ± 0.67	1.35 ± 0.48
	7	I think the game's designed environment is simple and user-friendly	21 (37.5)	30 (53.6)	5 (8.9)	0 (0.00)	0 (0.00)	1.29 ± 0.62	
	8	I think gamification technologies improve user satisfaction and appeal	37 (66.1)	15 (26.8)	4 (7.1)	0 (0.00)	0 (0.00)	1.59 ± 0.62	
Coherency	9	I think the game's media is in line with its subject matter	29 (51.8)	25 (44.6)	0 (0.00)	2 (3.6)	0 (0.00)	1.45 ± 0.68	1.39 ± 0.59
	10	I think the content's hierarchical structure is harmonious	27 (48.2)	23 (41.1)	4 (7.1)	2 (3.6)	0 (0.00)	1.34 ± 0.76	
	11	I think the game's language is appropriate for its subject matter	31 (55.4)	18 (32.1)	6 (10.7)	1 (1,8)	0 (0.00)	1.41 ± 0.75	
Esthetic and Consistency	12	I think the game's text can be used without tiring the eyes	29 (51.8)	19 (33.9)	8 (14.3)	0 (0.00)	0 (0.00)	1.38 ± 0.72	1.34 ± 0.64
	13	I think proper color balance is utilized in the creation of the game	24 (42.9)	27 (48.2)	4 (7.1)	1 (1.8)	0 (0.00)	1.32 ± 0.69	
Visual Aspects of Pictures, Text and Content	14	I think the density and balance of the shapes, images, and text were considered when designing the game	27 (48.2)	21(37.5)	3 (5.4)	5 (8.9)	0 (0.00)	1.25 ± 0.91	
	15	I think the font's color is appropriate	23 (41.1)	24 (42.5)	7 (12.5)	2 (3.6)	0 (0.00)	1.21 ± 0.80	1.28 ± 0.61
	16	I think the font size is suitable	26 (46.5)	21 (37.5)	7 (12.5)	2(3.6)	0 (0.00)	1.27 ± 0.82	
	17	I think the background color is appealing	25 (44.6)	22 (39.3)	8 (14.3)	1 (1.8)	0 (0.00)	1.27 ± 0.77	
	18	I think the font style and letter spacing are appropriate	24 (42.9)	24 (42.9)	8 (14.3)	0 (0.00)	0 (0.00)	1.29 ± 0.70	
	19	I think the images and legends are of sufficient quality	31 (55.4)	20 (35.7)	3 (5.4)	2 (3.6)	0 (0.00)	1.43 ± 0.75	
Technology and Multimedia Support	20	I think the game's user manual is appropriate	31 (55.4)	18 (32.1)	5 (8.9)	2 (3.6)	0 (0.00)	1.39 ± 0.80	1.49 ± 0.60
	21	I think instructional videos are used effectively for presenting the content, in addition to the sample questions	37 (66.1)	15 (26.8)	4 (7.1)	0 (0.00)	0 (0.00)	1.59 ± 0.62	
Educational Aspects of the Content	22	I think, playing the game has significantly improved my knowledge and practice of lasers	26 (46.4)	24 (42.9)	4 (7.1)	2 (3.6)	0 (0.00)	1.32 ± 0.76	1.26 ± 0.58
	23	I think after watching the videos and playing the games, I gained an understanding of the surgical procedure for treating oral lesions as well as other laser-based treatment modalities	19 (33.9)	29 (51.8)	6 (10.7)	2 (3.6)	0 (0.00)	1.16 ± 0.75	
	24	I think the information presented in the game greatly aids clinicians in choosing the type of laser and its settings	26 (46.2)	23 (41.1)	6 (10.7)	1 (1.8)	0 (0.00)	1.32 ± 0.74	
Attitude	25	I think, using lasers significantly increases the quality of oral lesions treatment	28 (45.9)	25 (44.6)	3 (5.4)	0 (0.00)	0 (0.00)	1.45 ± 0.60	1.40 ± 0.61
	26	I think lasers are less invasive than traditional methods	29 (51.8)	20 (35.7)	3 (5.4)	3 (5.4)	1 (1.8)	1.30 ± 0.93	
	27	I think, using lasers is more likely to result in patient cooperation and satisfaction than using conventional methods	34 (60.7)	16 (28.6)	4 (7.1)	2 (3.6)	0 (0.00)	1.46 ± 0.78	
Learning	28	I think that gamification has a major impact on learning the details and using the laser properly	28 (50.0)	22 (39.3)	4 (7.1)	2 (3.6)	0 (0.00)	1.36 ± 0.77	1.38 ± 0.66
	29	I believe that using new gamification technologies has a significant impact on educating the application of lasers in dentistry	29 (51.8)	23 (41.1)	2 (3.6)	2 (3.6)	0 (0.00)	1.41 ± 0.73	
**Total**									1.37 ± 0.46

Table 2 represents the difference between the questionnaire scores among genders and the correlation between the students' GPA and their satisfaction scores. The mean scores of female students were comparable regarding most of the presentations. However, female students rated Coherency (*p* = 0.019) and Learning perceptions significantly higher than male students. The Pearson correlation coefficient (r) between the participants' GPA and Design-related presentations, Performance-related perceptions, and Total Score were found to be 0.010, 0.05-, and 0.01-, respectively. Despite the low correlation coefficients observed between GPA scores and each measure, none of the correlations were statistically significant. These findings suggest no significant linear relationship between the students' GPA and satisfaction scores.

**Table 2:** The relationship between presentations’ mean score and participants’ gender

**Presentation**	**Gender**		***p* value**	GPA	***p* value**
	**Male**	**Female**		Pearson correlation coefficient	
	**Mean ± SD**^a^	**Mean ± SD**^a^			
**Design-related presentations**	1.24 ± 0.49	1.47 ± 0.44	0.087	0.010	0.941
1 Presentation	1.36 ± 0.55	1.54 ± 0.42	0.201	-0.04	0.74
2 Attraction	1.19 ± 0.47	1.44 ± 0.47	0.068	0.02	0.87
3 Coherency	1.14 ± 0.71	1.53 ± 0.48	0.019^*^	0.03	0.81
4 Esthetic and Consistency	1.21 ± 0.56	1.41 ± 0.68	0.257	-0.01	0.91
5 Visual Aspects of Pictures, Text and Content	1.22 ± 0.64	1.31 ± 0.60	0.621	0.002	0.99
6 Technology and Multimedia Support	1.31 ± 0.65	1.58 ± 0.57	0.122	0.04	0.73
**Performance-related perceptions**	1.17 ± 0.51	1.17 ± 0.52	0.68	0.05-	0.71
1 Educational Aspects of the content	1.12 ± 0.52	1.34 ± 0.61	0.188	0.69	0.69
2 Attitude	1.26 ± 0.65	1.44 ± 0.59	0.220	0.17	0.17
3 Learning	1.13 ± 0.79	1.51 ± 0.54	0.039^*^	0.97	0.97
Total Score	1.23 ± 0.48	1.44 ± 0.44	0.098	0.01-	0.90

^*^Significant difference between genders based on the result of student’s t-test (*p* < 0.05)

^a^The questions were ranked as follow: "Strongly agree = 2," "Agree = 1," "Neither agree nor disagree = 0," "Disagree = -1," "Strongly disagree = -2"

## Discussion

The findings of this pilot study revealed that last-year dental students at Mashhad Dental School positively evaluated the design and performance of ESKILLD. Students' gender and GPA did not affect their learning experience satisfaction level using ESKILLD.

Student satisfaction with the game's features and content may enhance learning through repetition, increasing motivation, and identifying complex concepts [3]. In several studies, students have reported that gamification enhances their interest in the course subject, knowledge acquisition, and retention, emphasizing the most critical aspect of the lesson for review [20]. Similar to the current study, high levels of user satisfaction and perception were reported in other studies using game-based learning for learning orofacial spaces [9], oral histology [6], factual knowledge [21], and clinical communication [13]. Tantawi et al. [22] reported that gamification significantly improved dental students' academic writing scores; however, their writing satisfaction and self-perceived improvement were modest. In their study, students' participation in academic writing was mandatory and might have affected their satisfaction. In contrast, participation in our study was voluntary. Despite the impossibility of comparing the studies due to the dissimilarity of the subjects and game designs, the participation condition may affect students' learning experience.

In a recent qualitative study, researchers investigated the most critical game elements from the perspective of medical students. The top three key elements identified were integration with instructional objectives, game rules, and rapid feedback [23]. In ESKILLD, educational videos were included, aligning with conventional theoretical laser course objectives and the instructional objectives. In addition, students answered various questions and played games related to the subject, which aimed to enhance information retrieval, strengthen information retention, and help them identify their weaknesses, something that is not feasible in a traditional learning environment [24]. Therefore, ESKILLD allows for both synchronous and asynchronous learning [25]. Students received immediate feedback on their correct or incorrect answers, which enabled them to estimate and track their progress [25]. Thus, the high user satisfaction with ESKILLD may be attributed to the incorporation of key gamification elements in the application's design. However, conducting a trial with a control group was not feasible due to the potential for the application being shared among the test subjects. Moreover, ESKILLD was utilized as a supplementary tool during the instructor's lecture. The study did not evaluate cognitive improvement. Furthermore, ESKILLD and the instructor's lecture were used as supplementary tools. As a result, it was not possible to assess the impact of ESKILLD on enhancing students' knowledge, especially in the absence of a control group.

Several studies have employed online gamification platforms (such as Kahoot! and Quizizz) as a learning tool. These platforms, which may or may not be teacher-oriented, are believed to improve learning by providing multiple-choice questions. In dentistry, Felszegly et al. [7] found that Kahoot! courses on medical and dental histology increased participant satisfaction. Nguyen et al. [3] found that using Kahoot! to learn microbiology and immunology improved dental students' attitudes towards the course, as well as their perceptions of their comprehension and retention of key concepts. Similarly, ESKILLD utilized multiple-choice questions, with 89.3% of participants reporting that gamification significantly enhanced their learning of laser information and applications. Nonetheless, while Kahoot! has a teacher-oriented design, ESKILLD can be used as a supplement to asynchronized material and contains other games.

There have been assessments of dental students' and practitioners' knowledge of laser applications in dentistry. According to a study conducted in Saudi Arabia, 76% of fourth-year dental students lacked adequate laser application knowledge [26]. In another investigation, most final-year dental students in Iran favored adding laser courses to the curriculum [27]. Dental clinicians also showed insufficient knowledge of laser application in dentistry [28, 29]. Sarabadani et al. [30] reported that using the mobile educational application Moodle to teach laser application in dentistry significantly increased dental students' knowledge compared to using a booklet. Consequently, portable, evidence-based interactive programs for learning laser applications in dentistry may enhance dental practitioners' understanding of this critical subject. In the current study, most students positively rated the educational aspects of the content of ESKILLD. Students have also reported familiarity with the surgical procedure for treating oral lesions (85.7%), and clinicians can use the game to select the laser type and setting (87.7%). Therefore, ESKILLD may be a valuable tool for students and practitioners to acquire or refresh their knowledge of lasers in dentistry knowledge. In addition, the content has been translated from Persian to English, Italian, and French, and the user can select the language upon entering the application; thus, students and clinicians worldwide can benefit from using this game.

Numerous studies have compared the effectiveness of digital learning methods to traditional lectures. Goob et al. [31] compared the satisfaction of dental students with Zoom conferences, Livestream, and prerecorded PowerPoint to traditional lecture formats. According to their findings, students were more satisfied with asynchronous learning. The widespread acceptance of asynchronized education is primarily because one can view the content again at a convenient time and place [32, 33]. However, asynchronized education lacks interaction, collaboration, and social exchange [34]. Therefore, numerous studies have concluded that combining traditional and digital instruction is the most effective method for achieving educational objectives and student satisfaction [35, 36]. Additionally, students may submit their questions via email. In the present study, ESKILLD was provided to students along with the conventional laser course, and students could consult with the professor regarding any questions. Due to the increasing popularity of digital education, however, additional research is required to compare the educational outcomes of using ESKILLD alone versus traditional lectures.

It is essential to take the gender variable into account when exploring learner attitudes and perceptions in serious games-assisted learning [37]. Men tend to be more analytical, confident, solitary, and assertive when playing serious games [38]. On the other hand, women tend to be collaborative, subjective, and reflective [38]. In the current study, although female students reported a higher coherency in ESKILLD content and better learning, both genders were similarly satisfied regarding the design-related and performance-related presentations. Furthermore, we hypothesized that students’ educational background could influence their learning experience. However, the current study participants’ GPA of the preceding five years did not influence their satisfaction. Therefore, ESKILLD seems to be an appealing game for various groups of students. Similarly, Sipiyaruk et al. [39] found that dental students from the United Kingdom and Thailand showed similar knowledge improvements after using a serious game learning environment for dental public health.

The current investigation had some limitations. The findings may not be generalizable because the study only included students from one dental faculty. In addition, due to the lack of research on educational games, it was impossible to evaluate the effects of game components. Like most other studies on gamification, our research concentrated on students, while limited research has been conducted on instructors' and faculty members' perceptions. Additionally, the application was designed for the Android operating system, thereby confining its usability to individuals using alternative operating systems. In this study, we have tried to overcome this limitation by a presentation session and providing a device with an Android operating system in the Laser department. However, due to accessibility limitations, students with iOS cell phones may not have enough time to appraise the game thoroughly. Therefore, future versions will consider other operating systems in the application design. Moreover, designing a trial with a control group was not possible due to the possibility of sharing the application between the test subjects. Furthermore, using qualitative feedback from the students on their learning experience in future studies would also help reveal positive and negative aspects of ESKILLD and areas of improvement to work on.

## Conclusion

In this study, ESKILLD, a new gamified laser application for dentistry, was developed. Sixth-year students reported high satisfaction with ESKILLD's design-related and performance-related presentations. The participants' positive attitude may reinforce the possibility of incorporating gamification in laser applications in dentistry courses. Furthermore, the GPA was not a significant factor in participants' satisfaction. Moreover, female and male students' satisfaction was comparable in most items and total scores. Therefore, ESKILLD may be appropriate for students from various educational backgrounds and genders.

## Data Availability

The datasets used and/or analyzed during the current study available from the corresponding author on reasonable request.

## References

[1] Papakoca K, Petrovski M (2021). Advantages of laser usage in dental implanology. KIJ.

[2] Arjunkumar R (2018). Awareness of laser dentistry among dentists in Tanjore-A survey. BPJ.

[3] Nguyen LM, Le C, Lee VD (2023). Game-based learning in dental education. J Dent Educ.

[4] Golshah A, Dehdar F, Imani MM, Nikkerdar N (2020). Efficacy of smartphone-based mobile learning versus lecture-based learning for instruction of Cephalometric landmark identification. BMC Med Edu.

[5] Deterding S, Dixon D, Khaled R, Nacke L, editors. From game design elements to gamefulness: defining" gamification". Proceedings of the 15th international academic MindTrek conference: Envisioning future media environments. 2011.

[6] Amir LR, Leonardy IC, Dewatmoko SN, Yanuar R, Suniarti DF, Idrus E (2023). Serious game as oral histology learning strategy for undergraduate dental students; crossover randomized controlled trial. BMC Oral Health.

[7] Felszeghy S, Pasonen-Seppänen S, Koskela A, Nieminen P, Härkönen K, Paldanius KM (2019). Using online game-based platforms to improve student performance and engagement in histology teaching. BMC Med Edu.

[8] Khoo E, Le A, Lipp MJ (2023). Learning games: A new tool for orthodontic education. Int J Environ Res Public Health.

[9] Arayapisit T, Pojmonpiti D, Dansirisomboon K, Jitverananrangsri K, Poosontipong D, Sipiyaruk K (2023). An educational board game for learning orofacial spaces: An experimental study comparing collaborative and competitive approaches, Anat. Sci Educ.

[10] Buajeeb W, Chokpipatkun J, Achalanan N, Kriwattanawong N, Sipiyaruk K (2023). The development of an online serious game for oral diagnosis and treatment planning: evaluation of knowledge acquisition and retention. BMC Med Educ.

[11] Aubeux D, Blanchflower N, Bray E, Clouet R, Remaud M, Badran Z (2020). Educational gaming for dental students: Design and assessment of a pilot endodontic-themed escape game. Eur J Dent Educ.

[12] Tuil N, Lescaille G, Jordan L, Berteretche MV, Braud A (2023). Implementation of game-based training in oral rehabilitation of edentulous patients in an undergraduate dental course. J Dent Educ.

[13] Lin CS, Yang CC (2023). Evaluation of a digital game for teaching behavioral aspects of clinical communication in dentistry. BMC Med Educ.

[14] Teerawongpairoj C, Tantipoj C, Sipiyaruk K (2024). The design and evaluation of gamified online role-play as a telehealth training strategy in dental education: an explanatory sequential mixed-methods study. Sci Rep.

[15] Akaltan KF, Önder C, Vural Ç, Orhan K, Akdoğan N, Atakan C (2023). The effect of game-based learning on basic life support skills training for undergraduate dental students. J Dent Educ.

[16] Wu JH, Du JK, Lee CY (2021). Development and questionnaire-based evaluation of virtual dental clinic: a serious game for training dental students. Med Educ Online.

[17] Prince M (2004). Does active learning work?. A Rev Res JEE.

[18] Alsadoon E, Alkhawajah A, Suhaim AB (2022). Effects of a gamified learning environment on students' achievement, motivations, and satisfaction. Heliyon.

[19] Almanasreh E, Moles R, Chen TF (2019). Evaluation of methods used for estimating content validity. Res Social Adm Pharm.

[20] Stoyanova M, Tuparova D, Samardzhiev K, editors. Gamification in 11th Grade Mathematics Lessons – One Possible Interactive Approach. Interactive Collaborative Learning; 2017 2017//. Cham: Springer International Publishing.

[21] Lemos M, Wolfart S, Rittich AB (2023). Assessment and evaluation of a serious game for teaching factual knowledge in dental education. BMC Med Educ.

[22] El Tantawi M, Sadaf S, AlHumaid J (2018). Using gamification to develop academic writing skills in dental undergraduate students. Eur J Dent Educ.

[23] Wang YF, Hsu YF, Fang KT, Kuo LT (2024). Gamification in medical education: identifying and prioritizing key elements through Delphi method. Med Educ Online.

[24] Larsen DP, Butler AC, Aung WY, Corboy JR, Friedman DI, Sperling MR (2015). The effects of test-enhanced learning on long-term retention in AAN annual meeting courses. Neurology.

[25] Saleem AN, Noori NM, Ozdamli F (2022). Gamification applications in E-learning: A literature review. Technol Knowl Learn.

[26] Al-Jobair A (2014). Dental laser education and knowledge among final year dental students at King Saud University in Riyadh, Saudi Arabia, Saudi. J Dent Res.

[27] Mehdipour M, Mortazavi H, Bahramian A, Haghighi Enayat N, Azari-Marhabi S (2020). The viewpoints of last-year dentistry students of Shahid Beheshti University on the application of lasers as an independent credit in the education of general dentistry. J Lasers Med Sci.

[28] Yadav S, Chaudhry S, Talwar S, Verma M (2018). Knowledge and practices of dental lasers among dental professionals in India: A survey-based study. J Dent Lasers.

[29] Bordea R, Lucaciu O, Câmpian RS (2016). Student's knowledge and opinion regarding the need of implementation of lasers in dental faculty curriculum. Hum Vet Med.

[30] Sarabadani J, Dehghani Tafti M, Labafchi A, Javan RA (2019). Comparing training of "Lasers in Dentistry" by two mobile-based and booklet approach training methods in dentistry students. J Mashhad Dent School.

[31] Goob J, Erdelt K, Güth JF, Liebermann A (2021). Dental education during the pandemic: Cross-sectional evaluation of four different teaching concepts. J Dent Edu.

[32] Kunin M, Julliard KN, Rodriguez TE (2014). Comparing face-to-face, synchronous, and asynchronous learning: postgraduate dental resident preferences. J Dent Educ.

[33] Grimes EB (2002). Student perceptions of an online dental terminology course. J Dent Educ.

[34] Ward ME, Peters G, Shelley K (2010). Student and faculty perceptions of the quality of online learning experiences. IRRODL.

[35] Jordan J, Jalali A, Clarke S, Dyne P, Spector T, Coates W (2013). Asynchronous vs didactic education: it’s too early to throw in the towel on tradition. BMC Med Edu.

[36] Al-Taweel FB, Abdulkareem AA, Gul SS, Alshami ML (2021). Evaluation of technology-based learning by dental students during the pandemic outbreak of coronavirus disease 2019. Eur J Dent Educ.

[37] Zhonggen Y (2019). A meta-analysis of use of serious games in education over a decade. Int J Comput Games Technol.

[38] Garber LL, Hyatt EM, Boya ÜÖ (2018). Constituting, testing and validating the gender learner profiles of serious game participants. Int J Educ Manag.

[39] Sipiyaruk K, Hatzipanagos S, Vichayanrat T, Reynolds PA, Gallagher JE (2022). Evaluating a dental public-health game across two learning contexts. Education Sciences.

