# Correction: The development of a serious game for laser applications in dentistry and the evaluation of dental students’ satisfaction

**DOI:** 10.1186/s12909-024-05721-7

**Published:** 2024-07-01

**Authors:** Maryam Khorasanchi, Melika Hoseinzadeh, Majid Khadem-Rezaiyan, Ali Kazemian, Ali Moradi, Javad Sarabadani

**Affiliations:** 1 Department of Endodontics, School of Dentistry, Mashhad University of Medical Sciences, Mashhad, Iran; 2 Dental Research Center, Mashhad Dental School, Mashhad University of Medical Sciences, Mashhad, Iran; 3 Department of Community Oral Health, Faculty of Dentistry, Mashhad University of Medical Sciences, Mashhad, Iran; 4 Medical Sciences Education Research Center, Mashhad University of Medical Sciences, Mashhad, Iran; 5 Department of Community Medicine, Faculty of Medicine, Mashhad University of Medical Sciences, Mashhad, Iran; 6 Orthopedic Research Center, Mashhad University of Medical Science, Mashhad, Iran; 7 Bone and Joint Research Laboratory, Ghaem Hospital, Mashhad University of Medical Science, Mashhad, Iran; 8 Oral and Maxillofacial Disease Research Center, School of Dentistry, Mashhad University of Medical Sciences, Mashhad, Iran

**Correction: BMC Medical Education (2024) 24:583**

10.1186/s12909-024-05563-3.

Following publication of the original article [1], the authors identified an error in the affiliation and author name of Majid Khadem-Rezaiyan.

The incorrect author name is: Majid Khadem Rezaeian.

The correct author name is: Majid Khadem-Rezaiyan.

The incorrect affiliation is:

Department of Community Medicine and Public Health, Faculty of Medicine, Mashhad University of Medical Sciences, Mashhad, Iran.

The correct affiliation is:

Medical Sciences Education Research Center, Mashhad University of Medical Sciences, Mashhad, Iran.

Department of Community Medicine, Faculty of Medicine, Mashhad University of Medical Sciences, Mashhad, Iran.

## References

[1] Khorasanchi. et al. BMC Med Educ (2024) 24:583. 10.1186/s12909-024-05563-3. 10.1186/s12909-024-05563-3 PMC11134726 38807167

